# The combinatorial activation of the PI3K and Ras/MAPK pathways is sufficient for aggressive tumor formation, while individual pathway activation supports cell persistence

**DOI:** 10.18632/oncotarget.6159

**Published:** 2015-10-19

**Authors:** Keyata N. Thompson, Rebecca A. Whipple, Jennifer R. Yoon, Michael Lipsky, Monica S. Charpentier, Amanda E. Boggs, Kristi R. Chakrabarti, Lekhana Bhandary, Lindsay K. Hessler, Stuart S. Martin, Michele I. Vitolo

**Affiliations:** ^1^ University of Maryland School of Medicine, Marlene and Stewart Greenebaum National Cancer Institute Cancer Center, University of Maryland School of Medicine, Baltimore, MD, USA; ^2^ Department of Pathology, University of Maryland School of Medicine, Baltimore, MD, USA; ^3^ Graduate Program in Molecular Medicine, University of Maryland School of Medicine, Baltimore, MD, USA; ^4^ Department of Pathology and Laboratory Medicine, Perelman School of Medicine, Univesity of Pennsylvainia, Philadelphia, PA, USA; ^5^ Department of Physiology, University of Maryland School of Medicine, Baltimore, MD, USA

**Keywords:** PI3K, PTEN, MAPK, breast cancer, dormancy

## Abstract

A high proportion of human tumors maintain activation of both the PI3K and Ras/MAPK pathways. In basal-like breast cancer (BBC), PTEN expression is decreased/lost in over 50% of cases, leading to aberrant activation of the PI3K pathway. Additionally, BBC cell lines and tumor models have been shown to exhibit an oncogenic Ras-like gene transcriptional signature, indicating activation of the Ras/MAPK pathway. To directly test how the PI3K and Ras/MAPK pathways contribute to tumorigenesis, we deleted PTEN and activated KRas within non-tumorigenic MCF-10A breast cells. Neither individual mutation was sufficient to promote tumorigenesis, but the combination promoted robust tumor growth in mice. However, *in vivo* bioluminescence reveals that each mutation has the ability to promote a persistent phenotype. Inherent in the concept of tumor cell dormancy, a stage in which residual disease is present but remains asymptomatic, viable cells with each individual mutation can persist *in vivo* during a period of latency. The persistent cells were excised from the mice and showed increased levels of the cell cycle arrest proteins p21 and p27 compared to the aggressively growing PTEN−/−KRAS(G12V) cells. Additionally, when these persistent cells were placed into growth-promoting conditions, they were able to re-enter the cell cycle and proliferate. These results highlight the potential for either PTEN loss or KRAS activation to promote cell survival *in vivo*, and the unique ability of the combined mutations to yield rapid tumor growth. This could have important implications in determining recurrence risk and disease progression in tumor subtypes where these mutations are common.

## INTRODUCTION

Although breast cancer (BC) is a phenotypically and molecularly heterogeneous disease, several common alterations to major signaling pathways have been found to result in accelerated cellular growth, differentiation, reduced cell death, and drug resistance, which collectively facilitate the step-wise progression seen in primary tumor development [1]. Among the most frequently dysregulated pathways in BC are the phosphatidylinositol 3-kinase (PI3K) pathway and the Ras/MAK pathway. Overactivation of the PI3K pathway has been seen in greater than 70% of tumors from patients with invasive BCs. Frequent mutations leading to PI3K pathway activation include PIK3CA mutations, PIK3CA copy number gain, PTEN loss, and AKT activation [2]. In addition to PI3K pathway activation, there is a large body of literature validating the important role for the Ras/MAPK pathway in BCs. Although activating mutations in the canonical Ras/MAPK pathway are infrequent in BCs (2-10%) [3–6], Ras/MAPK activity is commonly aberrantly stimulated *via* several independent mechanisms, including overexpression of receptor tyrosine kinases and loss of negative MAPK pathway regulators [7]. Elevated ERK1/2 activity (phosphorylation), a major effector of the Ras/MAPK pathway, has been observed in 50% of primary breast tumors as compared to adjacent normal tissue [8], and ERK phosphorylation has also been shown to be elevated in breast tumor cells capable of metastasis [8, 9].

The PI3K and Ras/MAPK pathways demonstrate a high level of signaling crosstalk, and accumulating preclinical data, in both cancer cell lines and murine models, suggest that concurrent inhibition of both pathways may successfully prevent cancer progression [10–13]. In order to block the proliferative and survival signals misregulated by PI3K and/or Ras/MAPK pathway activation, a reasonable approach may be to simultaneously inhibit both with small molecule inhibitors. However, these approaches are associated with high levels of toxicity to normal tissues, which require activation of at least one of these pathways for cell survival [14]. Therefore an improved understanding of the cross-talk and feedback mechanisms between the PI3K and Ras/MAPK signaling pathways is critical in order to develop effective targeted therapies with a tolerable toxicity profile.

BBC is an aggressive BC subtype associated with lower disease-free survival and higher risk of relapse that disproportionately affects African American patients [15–17]. This BC sub-type represents a major clinical challenge due to high mortality and limited target treatment options since a majority of BBCs are also typically triple-negative (TN) [3, 17–22] and patients with this BC subtype do not benefit from current targeted hormonal therapies. The major negative regulator of the PI3K pathway, PTEN, is lost or its expression is decreased in over 50% of all BBC cases [15, 23–25]. Additionally, gene amplifications of KRAS (32%), BRAF(30%), and EGFR (23%) are common to human BBCs [3] and BBC cell lines and tumor models have been shown to exhibit an oncogenic Ras-like gene expression signature [10]. To begin to elucidate how the PI3K and Ras/MAPK pathways could influence basal-like cell tumorigenesis, we created a model system using the human non-tumorigenic, mammary epithelial cell line, MCF-10A. The MCF-10A cells are well-suited for these studies since gene expression profile analyses have shown MCF-10A cells to cluster closely with the BBC sub-type and reflects the clinical “triple-negative” tumor type [26–28]. Using the MCF-10A cells also eliminates the confounding effects of additional mutations or genetic instability inherent in BC cell lines to allow a unique focus on the isolated effects of PI3K and Ras/MAPK pathway activation in the absence of widespread genomic instability. While previous studies have examined PTEN loss and Ras activation primarily in the context of accelerating the growth of existing tumor lines, there remains a need to understand how the activation of these individual pathways could contribute to cancer progression beyond that of initial tumor growth. We hypothesized that the activation of the PI3K pathway in combination with Ras/MAPK pathway, *via* PTEN loss and overexpression of activated KRAS, respectively, is sufficient to promote tumor initiation and progression in a non-tumorigenic cell line.

In this study, we demonstrate that the combination of PTEN loss and overexpression of activated KRAS yields a strikingly different phenotype *in vivo* that is not readily apparent with standard *in vitro* assays. The transplantation of PTEN−/−KRAS(G12V) cells into mice revealed that this mutation combination yields robust tumor formation, while cells bearing the individual mutations did not form tumors but could persist *in vivo* compared to the rapid disappearance of isogenic parental cells. Importantly, the surviving tumor cells with individual mutations could be recovered after long-term persistence, and upon reintroduction to growth-promoting conditions, were able to proliferate. These results highlight the potential for either PTEN loss or KRAS activation to promote tumor cell survival *in vivo* that could increase recurrence risk, and the unique ability of the combined mutations to yield rapid tumor growth that could influence tumor subtypes where these mutations are common.

## RESULTS

### PTEN loss cooperates with mutant KRAS(G12V) to promote growth factor independent and anchorage independent proliferation

**Figure 1:**
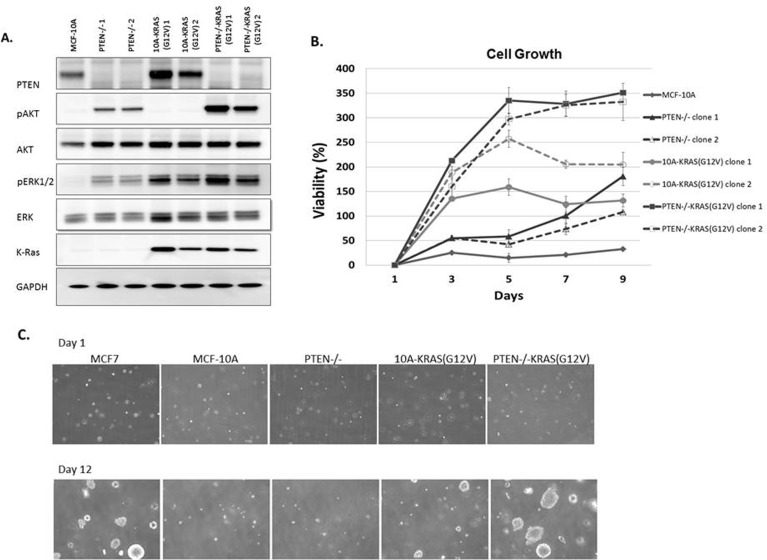
MCF-10A, PTEN−/−, 10A-KRAS (G12V) and PTEN−/−KRAS(G12V) pathway activation, viability, and colony formation in soft agar **A.** Western blot analysis of MCF-10A cells (lane 1), PTEN−/− clones 1and 2, (lanes 2 and 3), 10A-KRAS(G12V) clones 1 and 2 (lanes 4 and 5), and PTEN−/−KRAS(G12V) clones 1 and 2 (lanes 6 and 7) after plating in minimal assay media (1% charcoal dextran stripped FBS/DMEM/F12 devoid of growth factors) for 24h. **B.** Percent viability of MCF-10A cells, PTEN−/−, 10A-KRAS(G12V), and PTEN−/− KRAS(G12V) clones above initial day 1 after plating in minimal assay media. Percent viability was calculated by the following: ([(absorbance at day x/absorbance at day 1)-1] × 100). (*n* = 3, representative results from triplicate experiments). **C.** Colony formation in soft agar. Representative pictures at 4x are taken at the initial time of plating after agarose solidifies and after 12 days of incubation. MCF7 cells are used as a positive control.

Due to the multiple mutations within cancer cells, it is impossible to determine the direct signaling effect from a single oncogenic mutation without the consideration of possible compounding effects from additional mutations. To directly address this problem, we systematically introduced single oncogenic mutations in diploid non-tumorigenic, and genetically-stable MCF-10A mammary epithelial cell line. Since aberrant activation of the PI3K and Ras/MAPK pathways has been widely implicated in a variety of cancers, we created a model system to activate each pathway individually, starting with two of the most common mechanisms for pathway activation. PTEN, a regulator of the PI3K pathway, is the most commonly deleted tumor suppressor gene in cancers. To mimic the effects of PTEN loss, a MCF-10A-PTEN−/− mutant was previously generated and used for these studies [29]. Additionally, the Ras/MAPK pathway is dysregulated in approximately one-third of all human cancers. The majority of cancer-associated lesions within this pathway lead to constitutive activation of ERK signaling. ERK activation may occur from the overexpression of receptor tyrosine kinases, activation of mutations in receptor tyrosine kinases, sustained autocrine or paracrine production of activating ligands, *Ras*mutations and *B-Raf* mutations [30]. To activate the Ras/MAPK pathway, we expressed activated KRAS, KRAS(G12V), in MCF-10A parental cells and PTEN−/− cells. Protein immunoblots confirmed increased levels of pAKT and pERK, activation of the PI3K and Ras/MAPK pathways respectively (Figure 1A). pAKT levels are elevated in the PTEN−/− clones and pERK levels were elevated in the KRAS(G12V) clones. The clones were grown for 48h in media supplemented with only 1% charcoal dextran-stripped FBS, devoid of the exogenous growth factors normally incorporated in MCF-10A media indicating that the PI3K and Ras/MAPK pathways were constitutively activated. Interestingly, pERK levels were also elevated in the PTEN−/− cells, suggesting some cross-talk between the pathways. The pAKT levels in the PTEN−/−KRAS(G12V) clones were further increased over that of the PTEN−/− clones alone. pERK levels were also elevated in the PTEN−/−KRAS(G12V) clones, likely an additive effect since pERK levels increased in the both PTEN−/− and the 10A-KRAS(G12V) clones.

The MCF-10A cells require a specific combination of growth factors in order to proliferate. However, previous studies have shown that the biallelic deletion of PTEN, which recapitulates physiological PTEN expression loss, confers growth factor independent proliferation [29], a characteristic often associated with a transformed phenotype. As expected, in media devoid of growth factors, the MCF-10A cells were unable to proliferate and the PTEN−/− cells were able to slowly proliferate (Figure 1B). The addition of the mutant KRAS in the MCF-10A cells also conferred growth factor-independent proliferation. These results differ slightly from that of a previous study where the KRAS mutation (G12V) was knocked into a single allele of wild-type KRAS in the MCF-10A cells [31]. The single allelic knock-in does not confer EGF-independent growth. However, the MCF-10A cells used in this our study likely express multiple copies of KRAS(G12V) since they were retrovirally infected and have an increase in KRAS expression (Figure 1A), and thus could explain the different transformed phenotype of our KRAS(G12V) clones. Finally, the PTEN−/− cells expressing KRAS(G12V) strongly proliferate in the absence of growth factors (Figure 1B). The combined effect of PTEN loss and the addition of the oncogenic mutant KRAS overexpression induce a growth response greater than each mutation individually.

We subsequently cultured the cells in soft agar, to determine their ability to proliferate as anchorage-independent colonies, another characteristic associated with a transformed phenotype. Although PTEN loss, expression of KRAS(G12V), and the combination of mutations conferred growth factor-independent proliferation, only the KRAS(G12V) and PTEN−/−KRAS(G12V) cells were able to produce colonies (Figures 1C and S1). The KRAS(G12V) clones produced few, small colonies, similar to what has been shown with mutant HRAS(G12V) in immortalized human mammary epithelial cells [32]. However, the colonies observed from the PTEN−/− KRAS(G12V) were notably larger and occurred more frequently than those from the KRAS(G12V) clones (Figures 1C and S1).

### PTEN loss cooperates with mutant KRAS(G12V) to promote tumor development *in vivo*

**Figure 2:**
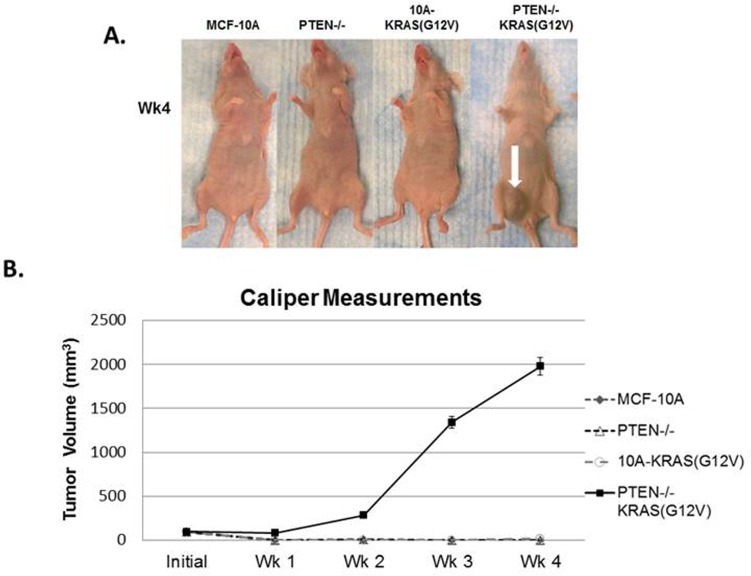
*In vivo* tumor growth of PTEN−/−KRAS(G12V) cells **A.** MCF-10A, PTEN−/−, 10A-KRAS(G12V), and PTEN−/−KRAS(G12V) cells were inoculated with Matrigel into the lower mammary glands of mice. The PTEN−/−KRAS(G12V) cells form large tumors in 4 weeks. No growth was detected by caliper measurements for the MCF-10A, PTEN−/−, or 10A-KRAS(G12V) cells. **B.** Graphical representation of the tumor size measured by calipers (*n* = 6).

Since the combination of PTEN loss and mutant KRAS(G12V) promotes robust anchorage-independent growth in soft agar, which is indicative of tumorigenicity, we hypothesized that MCF-10A cells with the combination of mutations would form primary tumors in mice. Since multiple clones of each of the PTEN−/−, 10A-KRAS(G12V), and PTEN−/−KRAS(G12V) performed similarly in our *in vitro* experiments, one clone from each set was prioritized for further *in vivo* studies. When injected into the mammary gland of female nude mice, PTEN−/−KRAS(G12V) cells formed large tumors, while the MCF-10A, PTEN−/−, and 10A-KRAS(G12V) cells failed to form palpable tumors (Figure 2A, 2B). Primary tumor growth of the PTEN−/−KRAS(G12V) cell line was detected as early as 1 week in the subdermal tissue of the inguinal nipple in NCR-nu/nu (nude) mice. The tumors resulting from inoculation of the PTEN−/−KRAS(G12V) cells grew rapidly between 1-4 weeks post inoculation and resulted in measureable tumor formation (6/6). Caliper measurements showed that the PTEN−/−KRAS(G12V) animal group examined 4 weeks post-inoculation exhibited an average primary tumor size of 1978.5mm^3^ (*n* = 10). Since the animals inoculated with the other lines did not display palpable tumors, caliper measurements could not be taken. While PTEN loss or KRAS(G12V) alone do not produce any detectable tumors, the combination of both mutations appear to synergize to promote robust primary tumor growth. Although the PTEN−/− cells have an increase in pAKT and pERK compared to the MCF-10A parental cells, they do not match the higher levels of pAKT and pERK in the PTEN−/−KRAS(G12V) cells (Figure 1A). This suggests that the elevated activation of both the PI3K and Ras/MAPK pathways is necessary and sufficient to promote tumor initiation and progression.

### PTEN loss and KRAS activation provide resistance to apoptosis

**Figure 3:**
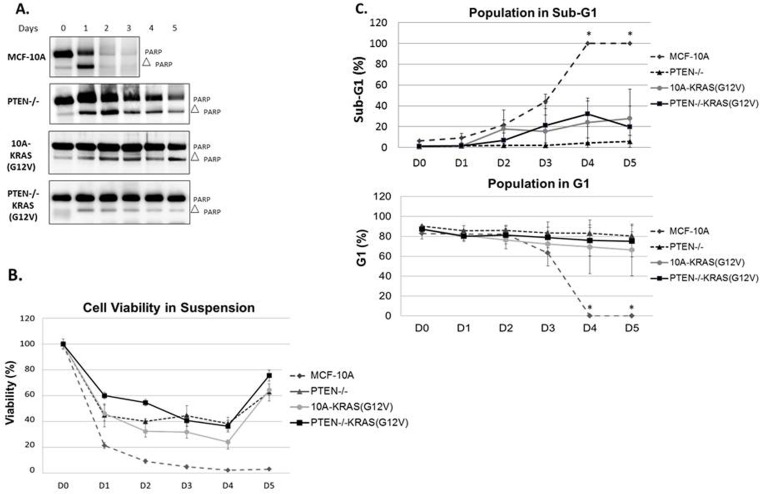
PTEN loss or KRAS activation provide resistance to apoptosis **A.** Cells were suspended in serum-free conditions over a series of days and harvested for Western blot analysis of PARP on the appropriate day. **B.** Viability of MCF-10A, PTEN−/−, 10A-KRAS(G12V) and PTEN−/−KRAS(G12V) cells in the same suspended, serum-free conditions over time. **C.** Graphical representation of percent of MCF-10A, PTEN−/−, 10A-KRAS(G12V) and PTEN−/−KRAS(G12V) cells in sub-G1 and G1 in the same suspended, serum-free conditions over time. *MCF-10A cells were dead by the fourth day and impossible to obtain the minimal 10,000 counts necessary for analysis. Since all were dead and fragmented via flow analysis, it is assumed that 0% of the population remained in G1 and 100% were in sub-G1.

The PTEN−/−, 10A-KRAS(G12V) and PTEN−/−KRAS(G12V) cells can all proliferate at increasing rates *in vitro* under growth factor-deprived culture conditions (Figure 1B). However, it is unlikely that only the differences in growth rates *in vitro* translates to the robust tumor growth of the PTEN−/−KRAS(G12V) cells *in vivo.* In addition to growth promotion, activation of the PI3K and/or the Ras/MAPK pathway can result in increased cell survival. We employed biochemical assays to next test the hypothesis that cells bearing the combination of PTEN loss and KRAS mutations also acquire a resistance to apoptosis, which could explain the dramatic increase in *in vivo* growth with the combined mutants. We examined the varying levels of PARP cleavage as a marker of apoptosis in each cell line grown over time in serum-free, suspended conditions. Although the MCF-10A cells do not significantly grow in minimal assay media, they also do not die (Figures 1B and S2); therefore serum was removed to further stress the cells for the apoptosis studies. However, MCF-10A cells do not reattach to tissue culture plates when split into serum-free conditions and remain in suspension. Thus, to maintain consistent conditions, all cells were suspended in serum-free media and assessed daily. By 24 hours, MCF-10A cells showed complete PARP cleavage which is distinctly different from the cells harboring the engineered mutations. As shown previously, PTEN−/− cells are more resistant to apoptosis than their parental counterparts [29]. Both the 10A-KRAS(G12V) and the PTEN−/−KRAS(G12V) cells show minimal PARP cleavage over the 5 days tested, indicated by the maintenance of full-length PARP (Figure 3A). Therefore, Western blot analysis reveals that overexpression of KRAS(G12V) may confer slightly more resistance to apoptosis than the loss of PTEN and the combination of mutations confers the most resistance.

Given the results of PARP cleavage apoptosis study, we conducted a colorimetric viability assay under the same suspended, serum-free conditions to confirm cell viability. The parental MCF-10A cells exhibited a drastic decrease in cell viability (Figure 3B), while the PTEN-/, 10A-KRAS(G12V) and PTEN−/−KRAS(G12V) each maintained a higher percentage of viable cells. The MCF-10A cells displayed a significant decrease in cell viability in 24 hours, with only 20% remaining viable. By comparison, at 24 hours the PTEN−/− and 10A-KRAS(G12V) cells were approximately 45% viable, while the PTEN−/−KRAS(G12V) cells were 60% viable. Even after 5 days, an average of 63% of PTEN−/−, 64% of 10A-KRAS(G12V), and 76% of PTEN−/−KRAS(G12V) were viable providing supporting evidence of enhanced survival. Activation of AKT and ERK was examined by Western blot analysis over 5 days under the same experimental conditions. Western blot analysis confirmed the increased levels of both pAKT and pERK with each mutation over time (Figure S3).

Due to increased resistance to apoptosis and viability observed in the mutant cell lines, we sought to determine with flow cytometry whether the cells were growth arrested or maintaining a balance between cellular proliferation and apoptosis to possibly help explain the differences observed in growth *in vivo* (Figure 2). Under the same suspended, serum-free conditions, samples were collected every 24 hours and fixed for flow cytometry analysis. Within 24 hours, 47% of the MCF-10A population had undergone apoptosis and were in sub-G1. However, the surviving PTEN−/− and 10A-KRAS(G12V) cells remained in the G1 portion of the cell cycle (Figure 3C) with no significant accumulation of cells in either S, G2, or G2/M over time (data not shown). The majority of the PTEN−/−KRAS(G12V) cells also remained in the G1 portion of the cell cycle, but after approximately 3 days had a minor decrease in the percentage of cells in the G1 with a simultaneous increase in the percentage of cells in G2 (Figure S4), indicating that these cells are likely slowly cycling even under these harsh conditions.

### PTEN loss and KRAS activation promotes reversible cell senescence

**Figure 4:**
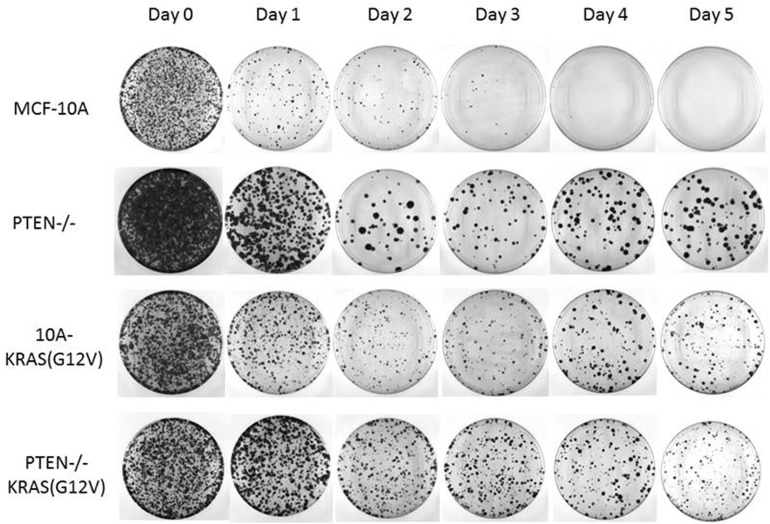
PTEN loss and KRAS activation promotes reversible cell cycle arrest Cells were suspended in serum-free media for the indicated time and returned to growth-promoting conditions (normal MCF-10A growth media and tissue culture treated dishes) for 10 days. The resulting colonies were fixed and crystal violet stained.

The viability and flow cytometry results revealed that the PTEN−/− and 10A-KRAS(G12V) were still viable and arrested in G1. While most of the PTEN−/−KRAS(G12V) cells were also viable and arrested in G1, a small population was determined to be in G2. To determine if the arrested cells would be able to re-enter the cell cycle and proliferate, they were placed back into growth-promoting conditions. Each cell line was again suspended in serum-free media and harvested every 24 hours. At each indicated time point, cells were collected, trypsinized to separate any clumping due to cell-cell association, and plated into normal growth media. The replated cells were then allowed to grow for 10 days. Subsequently, cells were formalin fixed and stained with crystal violet at representative time points. As expected, the MCF-10A cells had a significant reduction in the number of colonies formed over time, which could be attributed to their increased rate of death (Figure 4). However, the small population that was able to survive in these challenging conditions was also able to re-enter the cell cycle, as shown by the stained colonies on days 1, 2, and 3. The PTEN−/−, 10A-KRAS(G12V) and PTEN−/−KRAS(G12V) cells were all able to re-enter the cell cycle and proliferate once replated in growth promoting conditions over the entire time course (Figure 4). The later time points when the PTEN−/−KRAS(G12V) cells are likely to be slowly cycling (beginning after day 3) (Figure S4), it is reasonable to assume that the growth after replating is, at least in part, due to the cells which may already be actively, albeit slowly, growing. However during the initial 3 days, the PTEN−/−KRAS(G12V) cells accumulate in G1, yet cells harvested at the early time points also form colonies. The majority of the PTEN−/− and 10A-KRAS(G12V) cells accumulate in G1 without any significant accumulation in the other phases of the cell cycle, indicating the majority of cells are arrested in G1, yet, upon replating have a similar ability to regrow. It is important to note that the colony size differences between PTEN−/− and the other experimental cell lines is not due to growth rate differences since proliferation assays performed under the same growth conditions confirmed similar growth rates of the experimental cell lines (Figure S5). However, since single cell suspensions of PTEN−/− cells are known to have a greater ability to form aggregates, it is possible that the PTEN−/− cells quickly aggregated before replating [33].

The colony formation assay demonstrates that survival and viability in cells with PTEN loss and/or KRAS activation correlates strongly with the efficiency of colony formation. These mutations promote cellular arrest at the G1 checkpoint promoting survival until the cells are re-exposed to growth-promoting conditions, at which time they are capable of re-entering the cell cycle to continue to proliferate.

### PTEN loss or KRAS activation promotes a persistent phenotype *in vivo*

We initially observed that the PTEN−/−KRAS(G12V) cells formed tumors in mice and therefore these cells must maintain an increased proliferation rate and/or decreased apoptosis rate over the individual mutant cell lines. However, *in vitro* biochemical analysis could not determine major differences that would account for the strong distinction between the individual mutations and the combination for *in vivo* tumor growth. Therefore, these cells were monitored carefully with *in vivo* bioluminescence imaging. The use of bioluminescent optical imaging technology provides novel insight that exceeds traditional, non-luciferase human xenograft models. This technique has shown that human epithelial tumor cells that are transplanted, either under the skin or into the organ type in which the tumor originated, can be monitored or tracked as the disease progresses. In this report, the MCF-10A, PTEN−/−, 10A-KRAS(G12V), and PTEN−/−KRAS(G12V) cells were stably infected with a retroviral luciferase vector. Resultant high-expressing luciferase clones were injected subcutaneously into mice and monitored over time.

**Figure 5:**
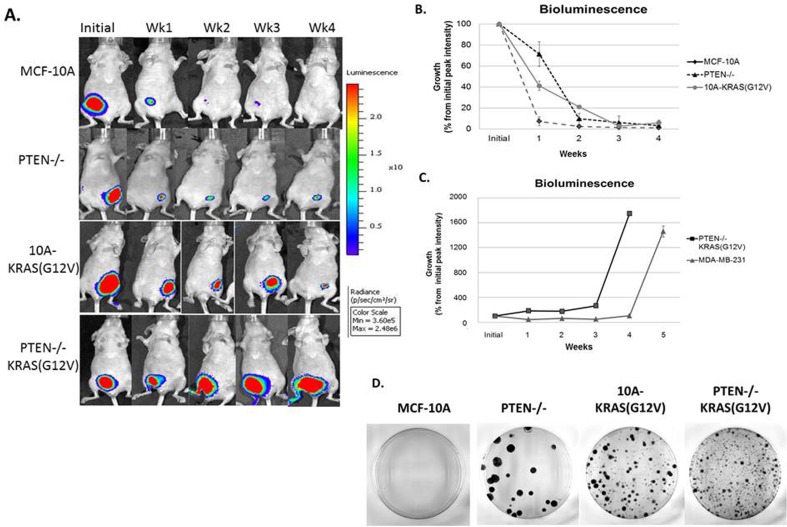
Persistence of PTEN−/− and 10A-KRAS(G12V) cells and primary tumor formation of PTEN−/−KRAS(G12V) cells in mouse xenografts **A.** A representative set of bioluminescence imaging from MCF-10A, PTEN−/−, 10A-KRAS(G12V) and PTEN−/−KRAS(G12V) over time. **B.** Graphical representation of the remaining bioluminescent signal measured initially and post injection. Percent signal is determined by subtracting background from the maximum signal on the appropriate day and normalizing the maximum signal minus background from day 0 on the same mouse (*n* = 10). **C.** Graphical representation of the bioluminescent signal of the PTEN −/−KRAS(G12V) and MDA-MB-231 cells, which represents the formation of a substantial tumor mass (*n* = 10). **D.** After 4 weeks in vivo, cells were harvested and dissociated for an *in vitro* crystal violet colony formation assay.

As predicted, the bioluminescence signal of animals inoculated with MCF-10A cells drastically declined over the observed time course and the signal resulting from PTEN−/−KRAS(G12V) cells continued to increase. However, the PTEN−/− and 10A-KRAS(G12V) cells persisted over time, a phenomenon that was undetectable with conventional caliper tumor measurements (Figure 2), but was now evident with bioluminescence (Figure 5A). At one week post inoculation, a bioluminescence signal was detected in each cell line, including the parental control MCF-10A cells. One week post inoculation, the signal from the MCF-10A cells was reduced to only 7%, while the PTEN−/− cell line maintained a bioluminescence of 71% of the initial peak intensity and the 10A-KRAS(G12V) was maintained at 41% (Figure 5B). This trend is maintained outward of 4 weeks post inoculation, and the cell persistence promoted by PTEN loss or KRAS activation *in vivo* becomes apparent. At four weeks, although the bioluminescence signal is diminished to 4% for the PTEN−/− and 6% for the 10A-KRAS(G12V) cells, it remains detectable, indicating survival of these cells. In addition to bioluminescence, caliper measurements were calculated (data not shown) and confirmed that there was a positive correlation between the bioluminescence intensity and the tumor volume in the PTEN−/−KRAS(G12V) cells. However, the viable PTEN−/− and 10A-KRAS(G12V) cells, detectable by bioluminescence, again remained undetectable by caliper measurements. These results emphasize the utility of bioluminescence for detecting persistent cells *in vivo*. Moreover, it is apparent that despite the non-tumorigenic phenotype of cells with either PTEN loss or KRAS activation, the dysregulation of either pathway contributes to survival, cellular viability, and persistence *in vivo*.

The bioluminescence of the PTEN−/−KRAS(G12V) cells continued to increase over the monitoring time course, reaching 18-fold over the initial bioluminescence in a period of 4 weeks (Figure 5A, 5C). MDA-MB-231 cells are typically classified by their invasive and tumorigenic phenotype, and when used as a comparison, the PTEN−/−KRAS(G12V) cells developed similarly robust tumors relative to this established tumorigenic BC cell line (Figure 5C). Both the MDA-MB-231 and the PTEN−/−KRAS(G12V) cell lines, on average, displayed measureable tumor growth between 4 and 5 weeks post inoculation. However, the PTEN−/−KRAS (G12V) cell line reached the experiments terminal endpoint at 4 weeks, sooner than the end point reached by the MDA-MB-231 cell line.

Due to the observed persistence of the PTEN−/− and 10A-KRAS(G12V) cells *in vivo*, we sought to determine the ability of the inoculated cells to re-enter the cell cycle and proliferate after persistence in mice. After 4 weeks *in vivo*, tumors from the PTEN−/−KRAS(G12V) cells were easily extracted, and we used the bioluminescent signal from the persistent cells to guide the tissue harvest of the PTEN−/− and 10A-KRAS(G12V) cells. After harvesting, the cells were dissociated and replated for colony formation. PTEN−/−, 10A-KRAS(G12V) and PTEN−/−KRAS(G12V) cells were all able to re-enter the cell cycle and proliferate once replated in growth promoting conditions after 4 weeks in mice (Figure 5D). The colony formation assay demonstrates that despite being unable to grow tumors *in vivo*, cells with either PTEN loss or KRAS activation can re-enter the cell cycle and proliferate under growth factor promotion.

### Immunohistochemical determination of proliferative and cell cycle arrest markers

After 4 weeks *in vivo*, the cells were also extracted for immunohistochemistry (IHC) staining and blindly scored by a comparative pathologist. As previously mentioned, the tumors from the PTEN−/−KRAS(G12V) cells were easily extracted, and we used the bioluminescent signal from the persistent cells to guide the tissue harvest of the PTEN−/− and 10A-KRAS(G12V) cells. However at this time point, there was no longer a bioluminescent signal at the site of the initial injection site of the MCF-10A cells indicating their disappearance, and therefore the MCF-10A cells could not be analyzed by IHC. We used anti-human mitochondrial protein to verify the location of the human cells and sections were analyzed for proliferation, apoptosis and cell cycle arrest using Ki67, caspase 3, p21, and p27 expression. A high index of Ki-67 and low caspase expression is commonly used as prognostic marker in breast cancer. Additionally since our *in vitro* results indicated that the PTEN−/−, 10A-KRAS(G12V), and PTEN−/−KRAS(G12V) significantly arrested in G1, we analyzed our tissue samples for p21 and p27, both known to regulate the G1/S checkpoint.

**Figure 6:**
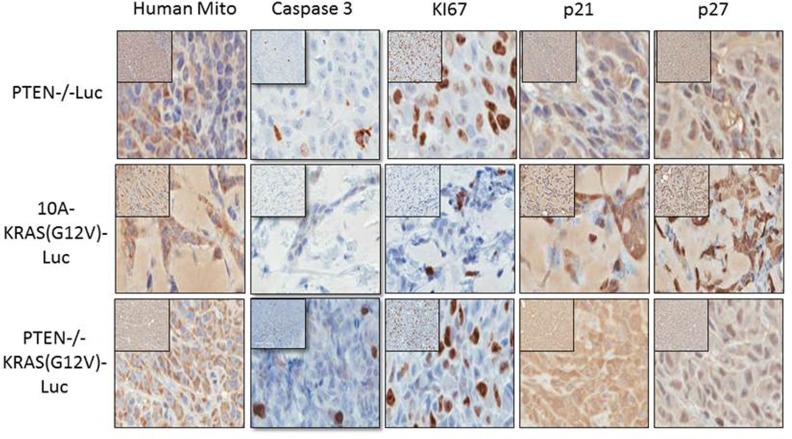
Immunohistochemistry of proliferative and cell cycle arrest markers in PTEN−/−, 10A-KRAS(G12V), and PTEN−/−KRAS(G12V) Representative images of immunostained samples for caspase 3, human mitochondria, Ki67, p21 and p27 Dark brown nuclei indicated positivity for p21 and p27.

The PTEN−/−KRAS(G12V) cell line (*n* = 6) displayed moderate to severe degrees of nuclear atypia, nuclear enlargement, variation in nuclear size, and the expression of Ki67 was high (75-100%) (Table 1 and Figure 6). By comparison, 51-75% of the PTEN cells (*n* = 6) and only 26-50% of the 10A-KRAS(G12V) cells (*n* = 6) stained positive for Ki67. The PTEN−/− and PTEN−/−KRAS (G12V) were minimally positive for caspase staining, while the 10A-KRAS(G12V) cells were negative for caspase. p21 and p27 were undetectable in the nuclei of the PTEN−/−KRAS(G12V) cells. In contrast, the PTEN−/− cells expressed insignificant levels of p21 but low levels of p27, and the 10A-KRAS(G12V) cells showed moderate positivity for both proteins.

**Table 1:** Pathological scoring of immunostained samples

	Human Mito	Caspase	Ki67	p21	P27
PTEN−/−Luc	(+)	(+)	(++)	(−)	(+)
10A-KRAS(G12V)-Luc	(+)	(−)	(+)	(++)	(++)
PTEN−/−KRAS(G12V)-Luc	(+)	(+)	(+++)	(−)	(−)

PTEN loss results in high proliferation markers, but also expression of caspase and the cell cycle arrest protein p27. Similarly, KRAS activation results in lower proliferation markers, no caspase expression, but both p21 and p27 are expressed by immunohistochemistry. Neither PTEN loss nor KRAS activation resulted in primary tumor development, suggesting that cells with either alteration do not confer rapid growth rates and are likely unable to overcome the challenge of growth arrest. Although the direct mechanism by which dormancy occurs is beyond the scope of this study, it is plausible that the loss of PTEN and KRAS activation could act as potential drivers of clinical tumor dormancy.

## DISCUSSION

Molecular profiling studies have confirmed that breast cancer (BC) represents four distinct subtypes (luminal A, luminal B, Her2+, basal-like) with different underlying biological aberrations [3, 10, 26] and that each major subtype has different prognostic outcomes in terms of patient survival. A recent, large-scale study of human breast tumors from the Cancer Genome Atlas Network, categorizes the common somatic and germline mutations, gene deletions and amplifications, hypermethylation, and protein up- and down regulation within BC subtypes of 510 tumors from 507 patients [3]. This database provides information regarding the frequency of alterations and their relationship to the BC molecular subtypes. Luminal/ER+ and Her2+ BCs had a higher incidence of PIK3CA mutations, over 49% and 42% respectively, than basal-like BCs (7%). However, the Her2+ and basal-like BCs had an increased incidence of PTEN loss, over 19% and 35% respectively. Although PIK3CA coding mutations are not common in basal-like BCs, this study determined the PIK3CA is amplified in over 49%. Even though only the most frequently mutated or lost components of the PI3K pathway were examined, it is clear that a large percentage of all BC subtypes have an activated PI3K pathway.

In addition to the activation of the PI3K pathway, the same study revealed high protein and phosphoprotein expression of both EGFR and Her2 within the Her2+ BC subtype, and both receptors have the ability to activate the Ras/MAPK pathway *via* GRB2 and SOS. Additionally, the BBCs had a variety of components of the Ras/MAPK pathway amplified including KRAS, BRAF and EGFR. In order to understand the combination of the activated PI3K and MAPK pathways and to begin to elucidate possible crosstalk and feedback mechanisms without confounding effects from mutated DNA repair proteins (ie. BRCA1/2 and p53), we used a model cell system with activation in both pathways in a non-tumorigenic, diploid, genomically stable cell line. We determined that the combination of PTEN loss and KRAS activation indeed activates the PI3K and Ras/MAPK pathways, respectively, *via* phosphorylation and thus activation of their downstream effectors, pAKT and pERK1/2. This combination leads to robust tumor growth in mice; tumor growth that surpassed that of the MBA-MB 231 breast cancer cells which are commonly exploited for xenografts. We report for the first time that PTEN loss and KRAS activation promotes cellular persistence, while the combination results in robust tumor formation *in vivo*. The IHC analysis of the tumors originating from the PTEN−/−KRAS(G12V) cells show high Ki67 staining, negative staining for the cell cycle arrest proteins, p21 and p27, and minor positivity for caspase; an expected staining profile for tumorigenic cells during growth. Both the PTEN−/− cells and the 10A-KRAS(G12V) cells similarly persist in the animal, but their IHC staining patterns differ. The PTEN−/− cells are more positive for Ki67 and less positive for the cell cycle arrest proteins than the 10A-KRAS(G12V) cells. This data suggests that the PTEN−/− cells are proliferating, albeit slower than the PTEN−/−KRAS(G12V) cells since Ki67 staining in the PTEN−/− cells is less than that of the PTEN−/−KRAS(G12V) cells, and that perhaps the PTEN−/− cells will form tumors over time. However, we have followed mice with the PTEN−/− xenografts for 2 years and these cells never form primary tumors. Additionally, the 10A-KRAS(G12V) cells stain more strongly positive for p21 and p27 (Table 1) which may loosely translate to more rapid cell cycle arrest than the PTEN−/− cells and thus an earlier disappearance of the cells and bioluminescent signal, but again, this is not the case. In fact, approximately 50% of the mice injected with the 10A-KRAS(G12V) cells form tumors with the average latency of 8 weeks post injection (data not shown). This is an unexpected result considering the lower levels of Ki67 and high levels of p21 and p27, but follows a previous pattern of Ras induced tumorgenicity. Previous studies using SV40/*hTERT* transformed primary human mammary epithelial cells (HMECs) expressing high levels of HRAS(G12V) formed tumors in 52% of the mice which were first apparent 7.5 weeks after initial injection [32]. Further analysis of more cell cycle regulator proteins, their phosphorylation status, and cellular locale is likely necessary for an accurate profile of cellular persistence *versus* proliferation.

Besides BCs, a significant body of evidence exists demonstrating the dual activation of the both the PI3K and Ras/MAPK pathway in a high proportion of human tumors, including lung [3, 34], ovarian [3], endometrial [35], prostate [36], colorectal [37, 38], bladder cancers [39], AML [35], and melanoma [35, 40], and it is clear that aberrations in the PI3K and the Ras/MAPK pathway coexist [36–38, 41, 42]. Reports show that PIK3CA activation mutations strongly desensitize RAS mutant cells to MEK inhibitors while depletion of PTEN confers resistance [43], and that KRAS activation mediates resistance to PI3K inhibitors [44, 45]. These studies provide a strong rationale for coexistence of the PI3K and Ras/MAPK activation and the necessity for identifying points of interactions and their consequences for better targeted therapies.

Inherent in the concept of cellular dormancy is the implication that cells retain the ability to become active under more favorable conditions. This study suggests that PTEN loss and Ras-activating mutations may contribute to cancer cell dormancy. There are multiple theories on potential mechanisms of tumor cell dormancy [46], such as loss of the aggressive phenotype through oncogene inactivation after the release of tumor cells from the primary tumor [47] and/or the need for additional genomic alterations to promote active growth post-seeding at a distant metastatic site [48]. Along these lines, it remains plausible that cells which have lost an aggressive phenotype or only acquired minimal mutations (ie. PTEN loss or KRAS activation), may be responsible for tumor cell dormancy. Additionally, dormant tumor cells are rare and difficult to locate with current technologies. Once found and isolated, further study is a serious challenge since dormant cells cannot be expanded without activating them. Our model system allows expansion *in vitro* for future *in vivo* studies in which the cells remain dormant and detectable. Importantly, current chemotherapies are ineffective against dormant tumor cells [49] since they are cell cycle arrested and resistant to systemic therapies targeted to active proliferation. Therefore, identifying mechanisms associated with the development, maintenance, and end of tumor cell dormancy are of great clinical importance. Along similar lines, it may be plausible to force aggressively growing tumor cells into dormancy to prevent tumor progression. Numerous studies have shown age retarding interventions, such as chronic rapamycin treatment, can decrease the incidence and/or severity of cancer [50, 51]. Rapamycin-induced lifespan enhancement of mice is, in part, due to attenuating cancer [51–56]. Rapamycin treatment causes continuous inhibition of mTOR kinase (mammalian target of rapamycin) in a variety of different tissues. While mTOR may not be the final molecule responsible for the anti-cancer effect, it remains an attractive target in cells which have lost PTEN since a major effector molecule of pAKT is mTOR. Future experiments will include chronic rapamycin treatment of mice bearing the PTEN−/−, 10A-KRAS(G12V), and PTEN−/−KRAS(G12V) cells to determine if rapamycin can eliminate dormant cells and/or delay the tumor formation from the aggressive PTEN−/−KRAS(G12V) cells.

We have shown that the dual activation of the PI3K and Ras/MAPK pathways, *via* PTEN loss and KRAS activation, is sufficient to promote robust tumor growth and thus, have developed a novel model system for elucidation of potential crosstalk and novel feedback mechanisms. Additionally, the novel finding is that PTEN loss or KRAS activation individually is sufficient to promote cellular persistence reminiscent of tumor cell dormancy, has potential implication for cancer dormancy and reoccurrence risk.

## MATERIALS AND METHODS

### Cell lines and cell culture

MCF-10A cells were purchased from ATCC (Manassas, VA) and maintained as previously described [29]. The creation of the PTEN−/− cells has been described previously [29]. The 10A-KRAS lines were constructed by retroviral infection of the oncogenic KRAS(G12V) gene. The pLXSN retroviral vector containing KRAS(G12V) was a generous gift from Dr. Ben Ho Park (Johns Hopkins University) [57]. Retroviral particles were generated using the AmphoPack-293 cells (Clontech) following manufacturer's instructions. MCF-10A cells were infected with the retrovirus in combination of 8 μg/ml polybrene and selected with 0.12 μg/ml neomycin after 48 hours. All luciferase expressing lines were infected with a luciferase retrovirus created using pMSCV-Luciferase PGK-hygro expression vector (Addgene, Cambridge, MA) [58]. Cells were maintained at 37°C with 5% CO_2_. MCF-7 and MDA-MB-231 cells were obtained from the American Type Culture Collection.

### Proliferation and survival assays

For initial growth assays, cells were seeded in triplicate at 2×10^4^ cells per well in a tissue culture treated 96-well dish in minimal assay media (DMEM/F12 without phenol red, 1% charcoal dextran-stripped FBS, and 100 units/ml penicillin-streptomycin, without exogenous growth factors). After 24 hours, CellTiter96 Aqueous One Cell Proliferation Solution (Promega, Madison, WI) was added, cells were incubated at 37°C for 2 hours, and the absorbance was determined at 490nm. Every two days the media was removed and replaced with fresh minimal assay media +/− CellTiter96. The absorbance after 24 hours was the initial growth (Day 1) for comparison with other days. Growth/viability was calculated as a percent above cell number on day 1 ([(absorbance at day x/absorbance at day 1)-1] × 100). All values are shown as mean ± SD of triplicate samples. For survival assays, cells were seeded in triplicate at 1.0×10^4^ per well in ultra-low attachment 96-well microplates in DMEM/F12 only. On each day, cell viability was quantified using CellTiter96. Growth/viability was calculated as a percent above cell number on day 0. All values are shown as mean ± SD from triplicate samples.

### Western blot analysis

Cells were harvested in immunoprecipitation lysis buffer [05mol/L Tris-HCl, pH7.4, 1.5mol/L NaCl, 2.5% deoxycholic acid, 10% NP-40, 10mmol/L EDTA] supplemented with protease inhibitor cocktail EDTA-free (Roche, Mannheim, Germany) and phosphatase inhibitor cocktail II (Calbiochem, LaJolla, CA). Primary antibodies anti-p-Akt(S473), anti-Akt, anti-p-ERK1/2, anti-ERK1/2, anti-p27, anti-p21, anti-PARP (Cell Signaling, Danvers, MA), anti-KRAS (EMD Millipore, Billerica, MA) and anti-GAPDH (Abcam, Cambridge, MA) were used at manufacturers' recommended dilutions.

### Colony formation in soft agar

7.5×10^3^ cells were cast in top layer medium composed of growth media and 0.6% SeaPlaque agarose (EMD Millipore). The cell mixture was plated in 6-well plates on top of a solidified layer of 0.8% agarose and growth media and allowed to solidify. The next day, 2 mls of growth media was added to the top of each well. Images were taken using an Olympus CKX41 inverted microscope equipped with the Olympus F-View II 12-bit CCD digital camera system and Olympus MicroSuite Five imaging software at 4x magnification on the initial day and 12 days after plating.

### Inoculation of breast cancer cells in nude mice

Eight-week-old female athymic nude-*Foxn1nu* mice weighing 18-23g, were obtained from NCI (Fredrick, MD) and fed food and water *ad libitum*. The mice were maintained in accordance with Institutional Animal Care and Use Committee procedures and guidelines under an approved protocol. Cell lines (1×10^6^ cells/ml) in 50% matrigel/PBS were injected subcutaneously into the 4th mammary gland on the ventral surface of the abdomen of athymic nude-*Foxn1nu* mice. Tumor volumes were measured by external caliper measurements weekly from the initial injection to 5 weeks. Tumors were measured along the two longest perpendicular axes in the x/y plane of each xenograft tumor to the nearest 0.1 mm with a digital caliper (Thomas Scientific, Inc.). Depth is assumed to be equivalent to the shortest of the perpendicular axes (y) and volume is calculated according to the formula: V = xy^2^/2, as is the standard practice for xenograft tumors. Signs of tumor ulceration were recorded during each measurement as an experimental endpoint. Mice were humanely euthanized at 4 weeks (terminal end point) as to not allow the maximum volume of the tumors (PTEN−/−KRAS(G12V) cells) to exceed 2cm^3^ (following IACUC procedures and protocols).

### Flow cytometry

For cell cycle analysis, cells were ethanol fixed and treated with RNase A (1mg/ml) and propidium iodide (PI) (20μg/ml). Cells were analyzed by a Becton Dickenson LSR-II at the Flow Cytometry Core Laboratory, CVD Immunology Group at the University of Maryland, Baltimore.

### Bioluminescence imaging

Luciferase expressing cells were injected subcutaneously into mice as above. At the indicated time points following injection, mice were injected intraperitoneally with Luciferin (150mg/kg, Perkin Elmer) and returned to their cages for 5 minutes to allow for biodistribution. Mice were anesthetized with 2% isoflurane gas and imaged at 5 minute intervals for the maximum photon emission. Total 60 photon flux (photons/sec) was calculated and corrected for tissue depth by spectral imaging using Living Image 3.0 software (Xenogen). Bioluminescence generated by the inoculated breast cell line in the experimental group was normalized to the initial signal and compared to the average light generated by the control animals. Bioluminescence was only detected in cells expressing the firefly luciferase gene, indicative of an active metabolism.

### *In vivo* cell harvesting and dissociation

Mice were humanely euthanized and tissue/tumor was excised. The tissue/tumor samples were minced into fine pieces with a sterile scalpel, suspended in a 1X solution of collagenase/hyaluronidase (Stem Cell Technologies, Vancouver, BC) and DMEM/12 and incubated at 37°C in a shaking incubator (200rpm) for 1h. Samples were centrifuged at 1000rpm for 5 min to pellet the cells. The cells were then cultured in MCF-10A growth media plus 0.1mg/ml hygromycin to select for luciferase expressing cells. After 14 days at 37°C and 5% CO_2_, the generated colonies were fixed with formalin and stained with crystal violet. This is a qualitative assay since we are unable to accurately determine the number of human cells plated after harvest, mincing, and dissociation.

### Immunohistochemistry and pathology

At four weeks post inoculation, 10 mice/cell line injected were sacrificed. The tumors or tissue were removed, fixed in formalin for 24 hours, embedded in paraffin wax, and serially sectioned (4-μm thick). All immunostaining was performed by Mass Histology Services (Worcester, MA). Anti-caspase 3 and Ki67 antibodies are supplied by Mass Histology. Anti-p21 and anti-p27 were purchased from Abcam and anti-human mitochondria antibody was purchased from EMD Millipore. Analysis and blind scoring was performed by Michael M. Lipsky, Ph.D., an independent comparative pathologist for the Department of Pathology.

### Ethics statement

All animal studies were performed following Institutional Animal Care and Use Committee procedures and guidelines at University of Maryland, Baltimore under an approved protocol.

## SUPPLEMENTARY MATERIAL FIGURES


