# Supplementary material for: The combinatorial activation of the PI3K and Ras/MAPK pathways is sufficient for aggressive tumor formation, while individual pathway activation supports cell persistence
Source: Oncotarget. 2015 Oct 19;6(34):35231–46. doi: 10.18632/oncotarget.6159 (PMC4742101; doi:10.18632/oncotarget.6159)
Supplement: Supplementary file 1 [file oncotarget-06-35231-s001.pdf]

The combinatorial activation of the PI3K and Ras/MAPK pathways is sufficient for aggressive tumor formation, while individual pathway activation supports cell persistence

Supplementary Material

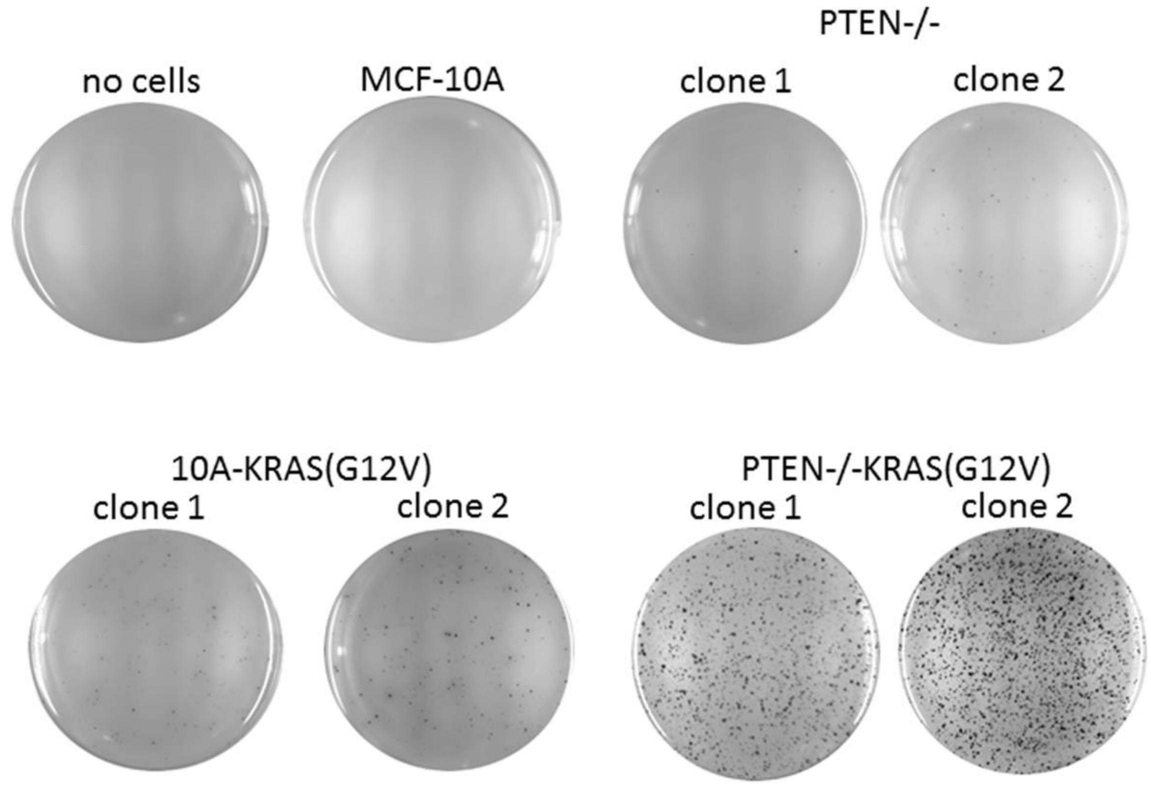

Supplemental Figure 1: MCF-10A, PTEN-/-, 10A-KRAS(G12V) and PTEN-/-KRAS(G12V) colony formation in soft agar. Images for entire 6-well of colony formation in soft agar. Cell were incubated and allowed to form colonies for 12 days, then stained with 0.1% iodonitrotetrazolium chloride (Sigma, St. Louis, MO) in PBS for 24hrs.

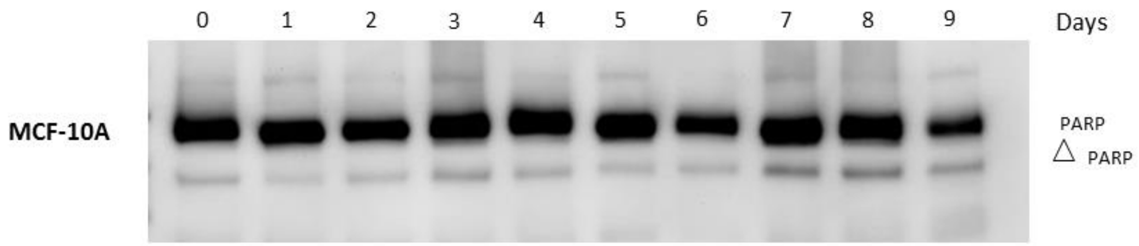

Supplemental Figure 2: MCF-10A PARP cleavage. Western blot analysis for PARP cleavage in MCF-10A cells plated in minimal assay media (1% charcoal dextran-stripped FBS) over time. These cells do not grow under these conditions (Fig. 1), but also do not significantly die over nine days.

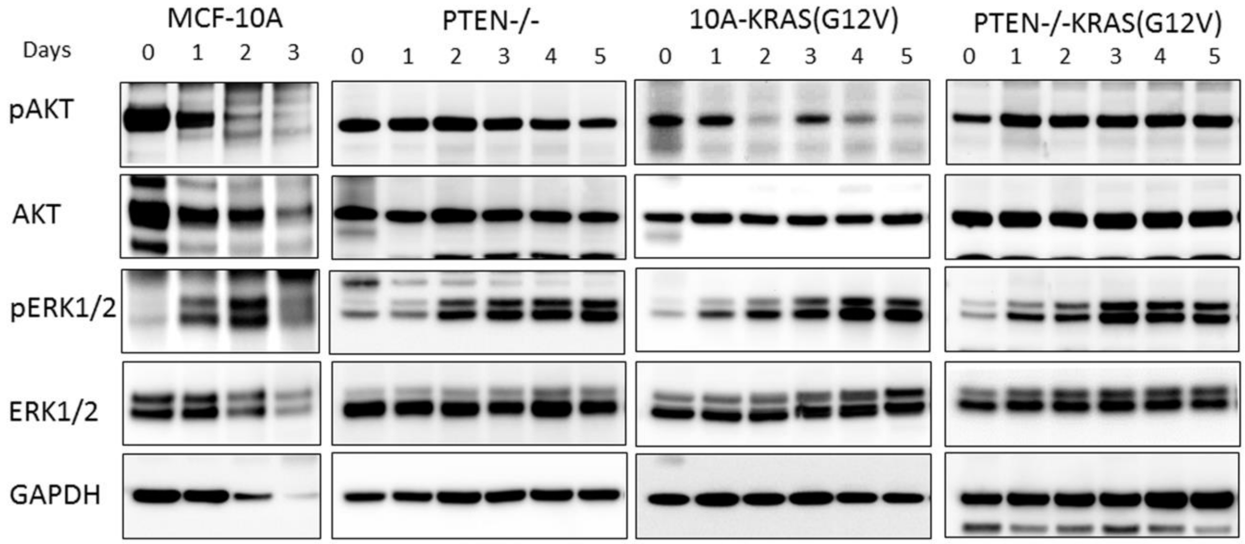

Supplemental Figure 3: Western blot analysis of pAKT and pERK. MCF-10A, PTEN-/-, 10A-KRAS(G12V), and PTEN-/-KRAS(G12V) pathway activation assessed daily after suspension in serum-fee media. The parental MCF-10A cells could only be taken out to 3 days due to massive cell death (Fig. 3).

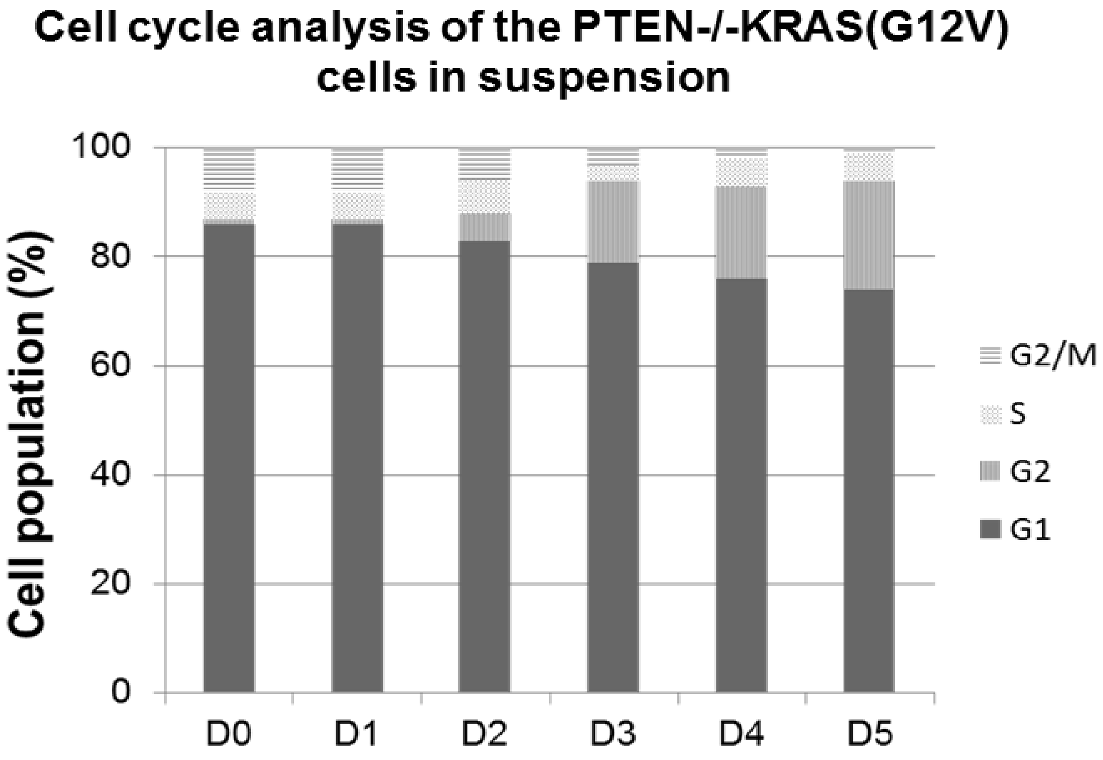

Supplemental Figure 4: Percentage of PTEN-/-KRAS(G12V) cell in the different phases of the cell cycle over time. Percent of cells accumulated in the G1, G2, S-phase, and G2/M. All values are shown as mean ± SD of triplicate samples.

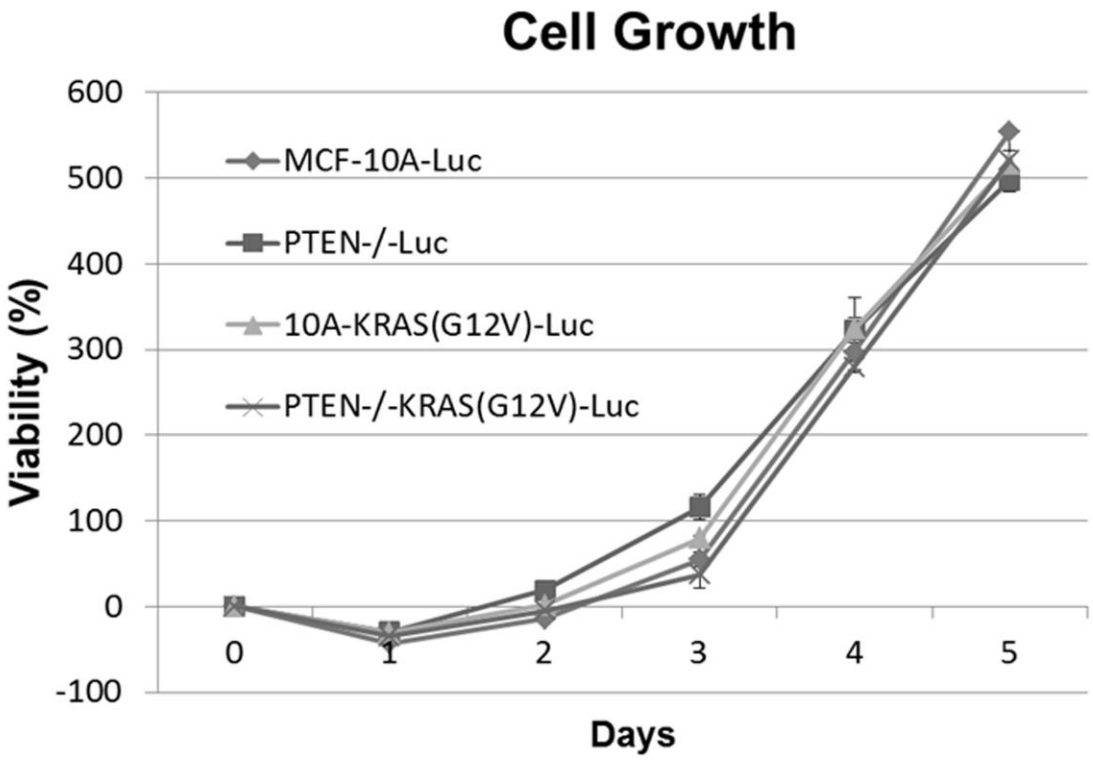

Supplemental Figure 5: Cell growth in normal culture conditions for the luciferase expression clones. MCF-10A-Luc, PTEN-/-Luc, 10A-KRAS(G12V)-Luc and PTEN-/-KRAS(G12V)-Luc were plated at equal densities ($1x10^3$ cells/well) in a 96-well plate. The CellTiter 96 assay was used as described above. All values are shown as mean ± SD of triplicate samples.
